# Case Report: A rare diffuse mitral valvular myxoma

**DOI:** 10.3389/fcvm.2024.1499553

**Published:** 2024-11-14

**Authors:** Dongqing Dou, Jun Wu, Wei Yuan, Haibo Wu, Qimin Wang

**Affiliations:** ^1^ Department of Echocardiography, Second Affiliated Hospital of Dalian Medical University, Dalian, China; ^2^ Department of Cardiac Surgery, Second Affiliated Hospital of Dalian Medical University, Dalian, China; ^3^ Department of Pathology, Second Affiliated Hospital of Dalian Medical University, Dalian, China

**Keywords:** cardiac tumor, myxoma, mitral valve, three-dimensional transesophageal echocardiography, valve replacement

## Abstract

Cardiac myxoma is a common benign tumor, however, myxoma extensively distributed on the mitral valve is rare and seldom reported. A patient who presented with exertional dyspnea and chest tightness was examined by transthoracic and transesophageal echocardiography successively. Multiple neoplasms on the mitral valve with moderate mitral regurgitation were found and were further proved to be a diffuse myxoma on the mitral valve by histology. Three-dimensional transesophageal echocardiography provided a precise evaluation of the mitral valve neoplasms, contributing to clinical decision-making.

## 1 Introduction

Cardiac myxoma is the most common primary benign tumor of the heart. However, a myxoma that is extensively attached to leaflets and sub-valvular structures is rare, the morphology and histological pathology of which are quite different from typical single or multiple myxomas on the cardiac valves or in the cardiac chamber. Three-dimensional transesophageal echocardiography (3D-TEE) can provide a detailed characterization of such diffuse lesions from a surgical perspective, contributing to accurate assessment and treatment decision-making for the patient.

## 2 Case presentation

A 51-year-old male was admitted to the hospital due to exertional dyspnea and chest tightness. The patient had no fever, dizziness, headache, amaurosis, nocturnal wheezing, or limb edema. He had a history of hypertension for more than 10 years without any special family history. Physical examination showed a full-systolic blowing murmur at the apex of the heart. The electrocardiogram (ECG) demonstrated normal sinus rhythm with a heart rate of 85 beats per minute.

**Figure 1:**
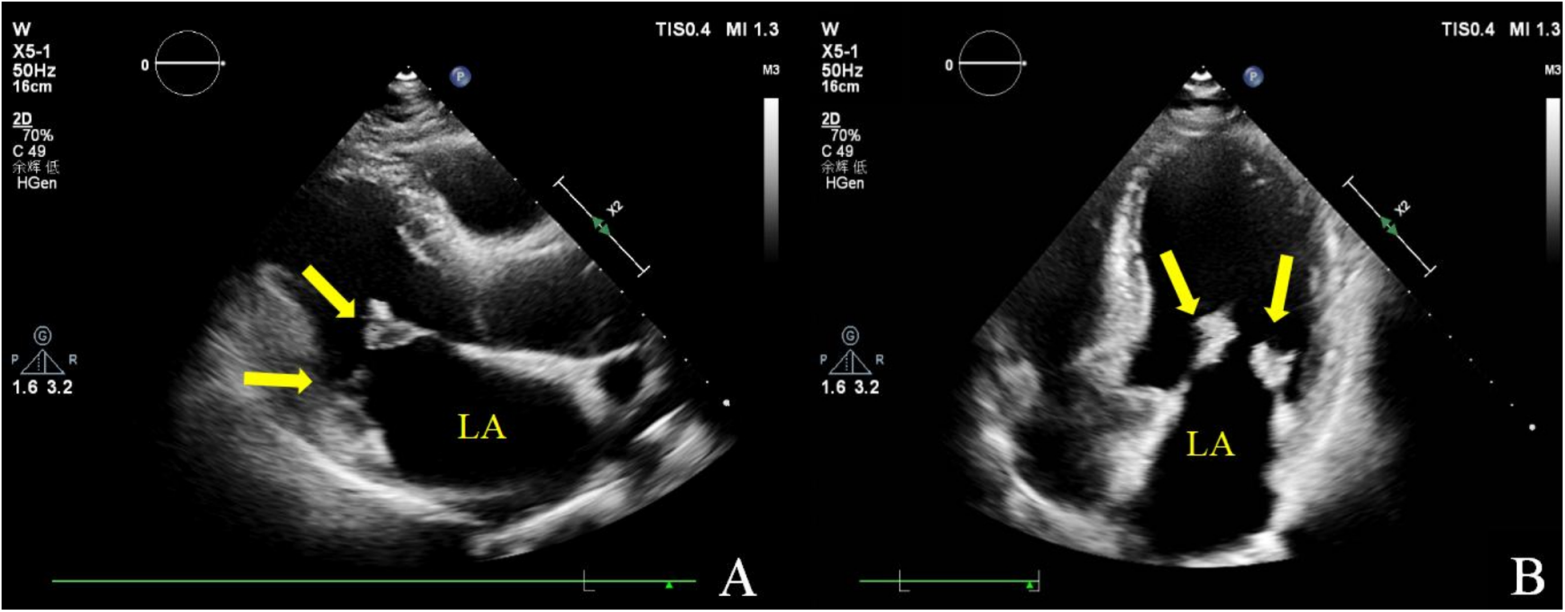
Transthoracic echocardiography. Multiple iso-echoic masses with irregular shapes were observed on the atrial sides of both the anterior and posterior mitral leaflets. Left ventricle long axis view **(A)** and apical four chamber view **(B)**.

**Figure 2:**
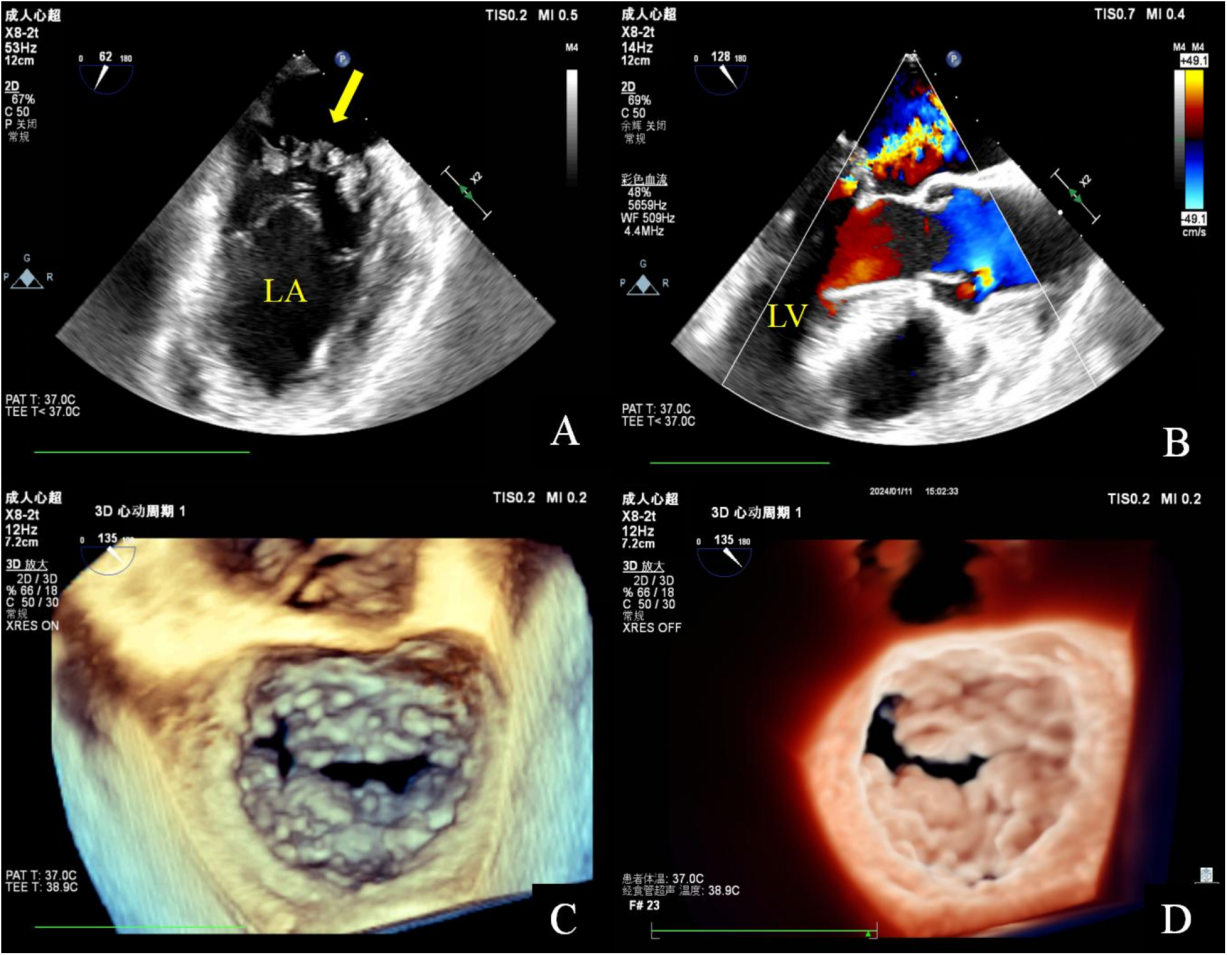
Transesophageal echocardiography. Mid-esophageal mitral commissural view: multiple irregularly shaped masses with a broad base attached to the anterior and posterior mitral leaflets. These masses are characterized by non-smooth surfaces, with an uneven internal echo **(A)**. Mid-esophageal long axis view: color doppler flow imaging indicated that the lesion has a soft and deformable texture, leading to moderate mitral regurgitation **(B)**. Three-dimensional transesophageal echocardiography: a diffuse distribution of granular additional echoes on the left atrial surface in two-thirds of the area of the anterior and posterior leaflets of the mitral valve, as well as the commissural cusps. The lesions merge with each other and are characterized by a wider base and a rough surface **(C,D)**.

**Figure 3:**
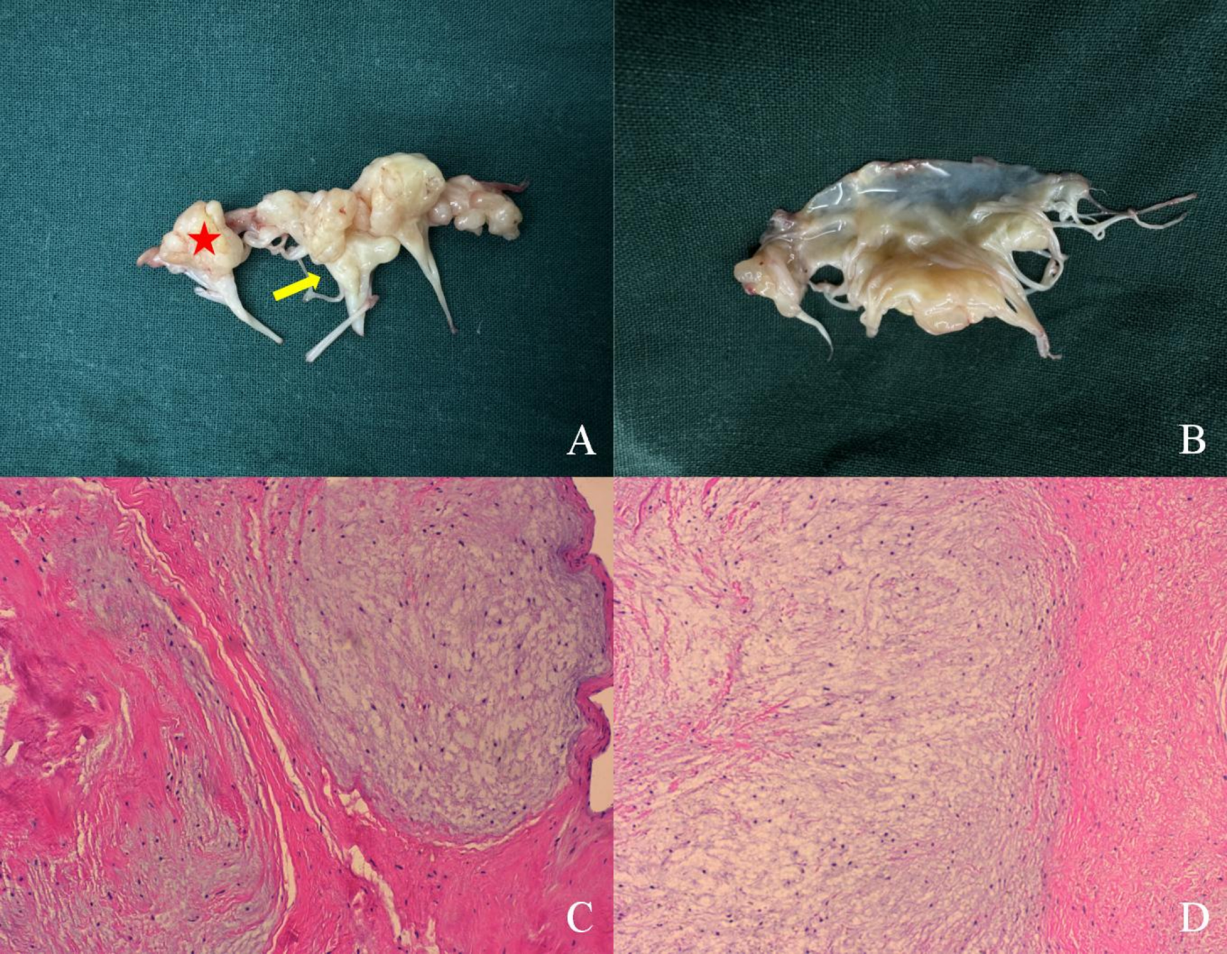
Pathological specimen. A cauliflower-shaped protrusion is prominently observed that is extensively adhered to the atrial surface. The myxoma extensively affects the mitral valve leaflets, commissural cusps (red star), and sub-valvular chordae tendineae (yellow arrow) **(A)**. No abnormalities are detected on the ventricular side of the leaflets **(B)**. Image under optical microscope (×10): the affected cells exhibit spindle or stellate morphology and are situated within an abundant mucinous matrix. The cellular distribution is sparse, exhibiting mild morphology, with no clear indications of nuclear division present **(C,D)**.

Two-dimensional transthoracic echocardiography (TTE) demonstrated left ventricular hypertrophy and left atrium enlargement. There were no segmental motion abnormalities. The patient’s left ventricular end-diastolic diameter (LVEDd) was 56 mm, his left ventricular end-systolic diameter (LVESd) was 40 mm, and his left ventricular ejection fraction (LVEF) was 57%. The anterior and posterior leaflets of the mitral valve were obviously thickened, especially on the edge area. Multiple iso-echoic neoplasms with irregular shapes were observed on the atrial sides of both the anterior and posterior mitral leaflets. The largest neoplasms were 14.6 mm × 12.0 mm on the anterior leaflet and 10.9 mm × 10.5 mm on the posterior leaflet, respectively (Figure 1). Color Doppler imaging showed moderate central mitral regurgitation (Supplementary Video S1). Laboratory tests showed no significant abnormalities in routine blood examination, coagulation, extractable nuclear antigen (ENA) antibody spectrum, liver function, and kidney function. Blood culture indicated no bacterial or anaerobic growth. A cranial CT scan suggested no signs of embolism in the brain. Computed tomography angiography (CTA) revealed the presence of calcified plaques on the thoracic and abdominal aortas, and also a lack of discernible indications of embolization. Infective and non-infective vegetations were ruled out based on these results. To further refine the neoplasm characterization, TEE was performed. TEE showed extensive thickening of the mitral valve, with multiple appendixes on the margins, with a smooth but irregular surface and a broad base attached to the anterior and posterior mitral leaflets (Figure 2). It seemed soft and deformed during valvular closure. Flickers could be seen with valve opening and closing, but without flail movement (Supplementary Video S2). Three-dimensional TEE revealed a diffuse distribution of granular neoplasms on the left atrial side of the mitral valve, especially two-thirds of the free edge area, as well as bilateral commissures. They were fused and the boundaries were unclear (Figure 2). The surface was cauliflower-shaped (Supplementary Video S3). There were no other valves involved in this case. Considering the risk of heart failure due to the valve disease and the risk of embolism, surgical treatment was performed. Intraoperative exploration showed that two-thirds of the area near the confluence margin of the anterior and posterior mitral leaflets were covered by diffuse tumor-like lesions. The commissural cusps and sub-valvular structures were involved as well, but there were no neoplasms on the left ventricular side of the leaflets. The lesions were merged with each other and characterized by a wider fundus and irregular shape. The granular structure partially extended toward the chordae tendineae. The impaired mitral valve was excised and submitted for histological examination and a microbiological culture. A prosthetic mechanical valve was replaced. An absence of bacterial proliferation was observed after a 48 h incubation for bacterial culture. The pathological examination showed a cauliflower-shaped protrusion approximately 2 cm × 1.5 cm × 1.2 cm in size on the excised mitral valve during the gross inspection (Figure 3). Under the light microscope, edema and mucinous degeneration were observed in the leaflet tissues, and there were spindle or stellate morphologic cells sparsely situated within an abundant mucinous matrix, which exhibited a mild morphology without abnormal nuclear division (Figure 3). The consultant pathologist suggested the diagnosis of a diffuse myxoma on the mitral valve with both leaflets, the commissural cusps, and the sub-valvular chordae tendineae extensively involved. The postoperative assessment indicated the prosthetic valve worked well (Table 1).

**Table 1:** Timeline.

Timepoint	Event
Half a month ago	The patient experienced exertional dyspnea and chest tightness
Day 1	The patient underwent transthoracic echocardiography in the outpatient department and was found to have a mitral valve mass, so he was admitted for treatment
Day 8	After completing the relevant examinations, the patient underwent transesophageal echocardiography
Day 9	Minimally invasive mitral valve replacement under general anesthesia and extracorporeal circulation
Day 13	The postoperative assessment indicated that the prosthetic valve worked well. The patient was discharged from the hospital

## 3 Discussion

Cardiac myxoma represents the most prevalent benign neoplasm of the heart, predominantly located within the atrium, occasionally observed on the cardiac valves and major blood vessels (1). This case demonstrated a diffuse myxoma that was extensively involved in the mitral valve and sub-valvular structures, the morphology and histological pathology of which were quite different from typical single or multiple myxomas on the cardiac valves or in the cardiac chamber. It needs to be differentiated from papillary elastofibroma (PFE), infective endocarditis, Libman–Sacks endocarditis, Barlow's disease, and primary or secondary malignant tumors (2).

PFEs are usually singular and commonly found in the aortic valve. PFEs show papillary fronds attached to the endocardium by a short pedicle. The tumor may have a sea anemone-like appearance with slight mobility. The histology shows a connective tissue core surrounded by a mucus-like matrix (3, 4), which is significantly different from the present case. In addition, the neoplasms on the leaflets and sub-valvular structures should be differentiated with vegetations caused by infective endocarditis or Libman Sacks endocarditis. Due to the patient's lack of infection history and symptoms and negative blood culture results, infective endocarditis was ruled out (5). Libman–Sacks endocarditis is characterized by inflammation and coagulation dysfunction on the basis of autoimmune diseases, leading to sterile vegetation formations, which are composed of platelets wrapped in a fibrous protein network (6). According to the patient's clinical manifestations and immunological examination results, Libman–Sacks vegetations were excluded before the operation. The histological results further confirmed our judgment. Barlow's disease also presents as myxomatous degeneration. However, a characteristic of Barlow's disease is excessive leaflet tissue billowing or prolapsing into the atrium in mid-late systole, instead of a granular neoplasm leaflet appearance (7). The most common malignant heart tumors include cardiac sarcomas, lymphomas, and melanomas. Malignant tumors can also exhibit irregular and sessile morphology on echocardiography due to the infiltration of adjacent structures. In cases where the history of malignancy is ambiguous, the use of cardiovascular magnetic resonance (CMR) or cardiac computed tomography (CCT) can assist in differentiating between benign and malignant tumors and in the detection of distant metastases (8–10). Although techniques such as CMR and CCT can clarify tumor infiltration by means of perfusion imaging, there are still limitations in the display of minute structures, and it is better to combine these with three-dimensional echocardiography for a multimodality imaging assessment (10, 11). In this case, the neoplasms on both the mitral leaflets did not have a pedicle and indeed did not possess the typical characteristics of a myxoma. However, pathology remains the gold standard for diagnosis. Combined with the gross inspection and histology, two experienced pathologists provided the diagnosis of diffuse mitral valvular myxoma.

For the management of the treatment of valvular masses, the extent and degree of the lesion, clinical symptoms, complications, and risk of embolism and recurrence need to be fully considered for conservative or surgical treatment (12–14). Thus, a precise preoperative echocardiography evaluation is necessary. Li et al. also reported a rare case of multiple myxomas of the mitral valve combined with typical embolic manifestations, with the intraoperative detection revealing multiple mucinous tumors attached to the mitral valve annulus, leaflets, and chordae tendineae. To avoid recurrence, the mitral leaflets and part of the mitral annulus, tendinous cords adherent to the neoplasms were removed, and prosthetic valve replacement was performed simultaneously (15). However, only two-dimensional TTE images were provided in this case report. In contrast to TTE, TEE demonstrates enhanced diagnostic sensitivity, mitigates the impact of pulmonary gas on image integrity, and offers distinct advantages in the detection of atypical locations and small neoplasms (11). In this case, we used 3D-TEE to perfectly demonstrate the surgical inspection before the operation. Three-dimensional transesophageal echocardiography can contribute to a comprehensive evaluation of the myxoma, including the size, number, range, border, surface, mobility, and sub-valvular structure involvement, and is thereby helpful for clinical decision-making (11, 13, 16).

In addition, embolization is the most common complication in patients with mitral valve mucinous tumors (17). An irregular surface and atypical pedicle attachment sites are strong risk factors for embolic events (1). Although the patient, in this case, has not yet exhibited any embolic symptoms, given the size and the irregular surface of the neoplasms, dilated cardiac chambers, and symptoms of heart failure due to chronic mitral regurgitation, surgical treatment was administered to correct his hemodynamics and eliminate the risk of embolism. In addition, the potential for multiple or ectopic recurrence should not be neglected (18, 19). The recurrence rate of cardiac myxoma has been documented to be 4%–7% in disseminated cases and 10%–21% in familial cases (20), therefore, regular follow-up is still necessary after surgery. Nevertheless, the majority of patients with myxoma have a favorable prognosis with symptomatic improvement following a surgical intervention (17). This case suggests that three-dimensional transesophageal echocardiography is an important tool for the comprehensive evaluation of cardiac myxoma and effective communication between the cardiac ultrasound and surgical teams.

## 4 Conclusion

Echocardiography can conveniently and quickly detect the presence of mitral valve neoplasms. Three-dimensional TEE provides a precise evaluation of mitral valve neoplasms, contributing to clinical decision-making.

## Data Availability

The original contributions presented in the study are included in the article/Supplementary Material, further inquiries can be directed to the corresponding author.
